# Characteristics and Types of Urolithiasis in the Eastern Region of Saudi Arabia: A Single-Center Retrospective Study

**DOI:** 10.7759/cureus.22913

**Published:** 2022-03-07

**Authors:** Ahmed Alasker, Saeed Bin Hamri, Yasser A Noureldin, Abdullah A Alsaghyir, Ghassan I Alhajress

**Affiliations:** 1 Urology, National Guard Hospital, Riyadh, SAU; 2 College of Medicine, King Saud bin Abdulaziz University for Health Sciences, Riyadh, SAU; 3 College of Medicine, King Abdullah International Medical Research Center, Riyadh, SAU; 4 Urology, Benha University, Benha, EGY

**Keywords:** urolithiasis, nephrolithiasis, stone analysis, renal stone disease, kidney calculi, renal calculi

## Abstract

Introduction

Urolithiasis is one of the most common conditions encountered in clinical practice with the prevalence increasing globally in the last few decades. Urolithiasis has been found to be more common in areas with a hot climate, such as Saudi Arabia. The aim of this study was to determine the characteristics and the types of urolithiasis most frequently found in the Eastern Region of Saudi Arabia.

Methods

This was a single-center retrospective cohort study based on data extracted from an electronic hospital information system (BESTCare) of all patients diagnosed with urolithiasis at King Abdulaziz Hospital, a tertiary care center in Saudi Arabia's Eastern Region. From January 2013 to December 2016, all adult patients aged 18 and up who presented with urinary calculi (renal and ureter) were included in the study.

Results

A total of 235 patients were reviewed, with a mean age of 48.52 years. Renal calculi were more prevalent in males (74.5%). Calcium oxalate was the predominant type (76%), followed by uric acid calculi (18%) and cystine calculi (4.8%). A small proportion (1.2%) was calcium phosphate calculi. The most frequently associated comorbidity was hypertension (17.9%). The majority (78.5%) had a stone removal through a ureteroscopy and 8.2% by percutaneous nephrolithotomy (PCNL). The mean stone size was 12.2 ± 9.91 mm, with a mean stone Hounsfield unit (HU) of 789.9. The mean urinary PH at stone incident was 6.77, and the mean creatinine level was 92.4mmol.

Conclusion

This study showed that males were more affected by urolithiasis, compared to females in the Eastern Region. Furthermore, calcium oxalate was the predominant type. These findings are consistent with the literature and they highlighted the necessity for further studies in this area, to provide insight into the pathophysiology and incidence of renal calculi for improving patient care.

## Introduction

Renal calculi are frequent conditions encountered in urological practice, with their prevalence increasing globally in the last decades [1]. Multiple factors have been described in the literature that could influence the prevalence of renal calculi; one of which is geographic variability. The prevalence of renal calculi has been reported to be much higher in areas with a hot climate [2-5]. The risk of developing renal calculi in adults is significantly higher in the Kingdom of Saudi Arabia (20.1%), compared to other Western countries (12% in Canada, and 13-15% in the USA), which may be explained geographically. With the hot climate, a decrease in body fluids through perspiration and inadequate fluid intake can raise the possibility of renal calculi formation substantially. Other factors that can contribute to the formation of renal calculi are hereditary factors, dietary habits, race, gender, age, occupation, and body weight [6,7]. 

There are many types of urinary calculi, and each has a special appearance and characteristics [8]. The most frequent type of renal calculi consists of calcium oxalate (75%) [9]. Identification of the substance structure of calculi is a fundamental advance in the metabolic assessment, treatment, and prevention of the disease. The aim of this study was to determine the characteristics and the types of renal calculi most frequently found in the Eastern Region of Saudi Arabia.

## Materials and methods

Study setting and design

This was a single-center retrospective cohort study, based on data extracted from an electronic hospital information system (BESTCare) of patients presenting with urolithiasis at King Abdulaziz Hospital a tertiary care center in the Eastern Region of Saudi Arabia. Ethical approval was obtained from the local Institutional Review Board (SP16/143/A)

Study participants and data collection

The study included all patients aged 18 years and older, who presented with urinary calculi (renal and ureter) from January 2013 to December 2016. Data were extracted from the electronic health records. Data included demographic information in terms of age, gender, body mass index (BMI), and stone type. Data were statistically analyzed to identify the characteristics and types of renal calculi. The calculi were retrieved after lithotripsy, spontaneously, PCNL (percutaneous nephrolithotomy) or after ureteroscopy. Exclusion criteria were applied to all patients with bladder calculi and pediatric patients due to the low incidence of renal calculi in that age group. 

Statistical analysis

The data were analyzed with the SPSS Statistics v. 21.0 (IBM Corp., Armonk, NY). Descriptive statistics (mean, standard deviation, frequency, and percentage) were calculated for all the variables. One-way analysis of variance (ANOVA) and a chi-square test were also performed to test the differences in selected variables. All tests with a p-value less than 0.05 were considered significant.

## Results

A total of 235 patients were reviewed, with a mean age of 48.52 years. In the Eastern Region, kidney calculi were more prevalent in males (74.5%). The majority of patients were overweight or obese and the mean BMI of 29.8 kg/m2. The most frequently associated comorbidity was hypertension (17.9%), followed by non-insulin-dependent diabetes mellitus (15.7%) and insulin-dependent diabetes mellitus (3.8%), and a small proportion (2.6%) had dyslipidemia (Table 1). 

**Table 1:** Incidence of renal stone according to age, gender, BMI, and comorbidities *BMI (body mass index), HTN (hypertension), NIDDM (non-insulin-dependent diabetes mellitus), IDDM (insulin-dependent diabetes mellitus), DLP (dyslipidemia).

Study variables	N (%)
Age in years (mean ± SD)	45.9 ± 14.0
Gender:	
Male	175 (74.5%)
Female	60 (25.5%)
BMI in kg/m2 (mean ± SD)	29.8 ± 6.56
Comorbidities:	
HTN	42 (17.9%)
NIDDM	37 (15.7%)
IDDM	09 (03.8%)
DLP	06 (02.6%)

**Figure 1:**
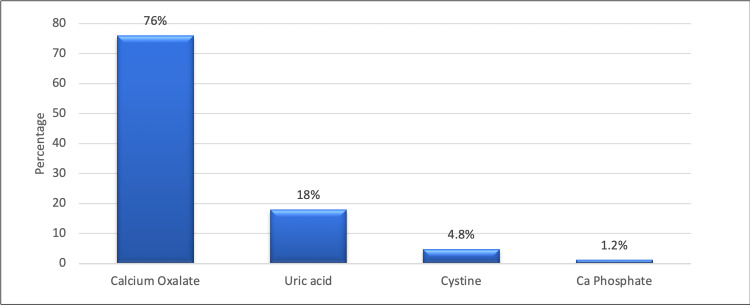
Distribution of the type of renal stone

Calcium oxalate calculi were the predominant type (76%) of all the calculi, followed by uric acid calculi (18%) and cystine calculi (4.8%). A small proportion (1.2%) was calcium phosphate calculi (Figure 1). 

Regarding the management, 13.4% of the patients had spontaneous stone passage with no intervention required. The majority (78.5%) had a stone removal through a ureteroscopy and 8.2% by percutaneous nephrolithotomy. The mean stone size was 12.2 ± 9.91 mm, with a mean stone Hounsfield unit (HU) of 789.9. The mean urinary PH at stone incident was 6.77, and the mean creatinine level was 92.4 mmol (Tables 2-3).

**Table 2:** Prevalence of renal stone by location *UC (upper calyx), MC (middle calyx), LC (lower calyx).

Variables	N (%)
UC	63 (26.8%)
MC	76 (32.3%)
LC	115 (48.9%)
Renal pelvis	51 (21.7%)
Upper ureter	30 (12.8%)
Mid ureter	06 (02.6%)
Lower ureter	09 (03.8%)
PH (mean ± SD)	6.77 ± 6.34
Stone burden in mm (mean ± SD)	12.2 ± 9.91

**Table 3:** Association between the type of renal stone among the baseline and clinical characteristics of the patients * IDDM (insulin-dependent diabetes mellitus), NIDDM (non-insulin-dependent diabetes mellitus), HTN (hypertension), DLP (dyslipidemia). BMI (body mass index), Hb (hemoglobin§ P-value has been calculated using Fischer exact test. ‡ P-value has been calculated using the one-way ANOVA test. ** Significant at p<0.05 level.

Factor	Ca oxalate N (%)	Uric Acid N (%)	Cystine N (%)	Ca Phosphate N (%)	P-value ^§^
Total No	127	30	8	2	
Percentage (%)	76%	18%	4.8%	1.2%	
Gender					
Male	102 (80.3%)	27 (90.0%)	0	02 (100%)	<0.001 **
Female	25 (19.7%)	03 (10.0%)	08 (100%)	0	
Comorbidities					
IDDM	05 (03.9%)	03 (10.0%)	0	0	0.407
NIDDM	19 (15.0%)	07 (23.3%)	0	0	0.446
HTN	17 (13.4%)	09 (30.0%)	0	0	0.090
DLP	03 (02.4%)	02 (06.7%)	0	0	0.446
	Mean ± SD	Mean ± SD	Mean ± SD	Mean ± SD	P-value ^‡^
Age in years	48.2 ± 13.3	44.9 ± 11.9	31.9 ± 3.04	55.0 ± 19.8	0.004 **
BMI in kg/m2	29.8 ± 6.06	31.7 ± 4.48	31.7 ± 5.70	30.3 ± 0	0.386
PH	6.23 ± 0.76	5.57 ± 0.58	7.50 ± 0.89	7.00 ± 1.41	<0.001 **
Stone size in mm	10.5 ± 7.55	17.4 ± 11.5	10.2 ± 8.15	9.00 ± 0	0.002 **
Creatinine clearance	91.3 ± 26.2	87.4 ± 14.5	85.5 ± 14.8	--	0.778
Hb (%)	13.4 ± 1.94	13.7 ± 2.05	12.0 ± 0.00	--	0.496

The most frequent location of the stone was in the left ureter (52.3%) (Table 2). The uric acid calculi were significantly associated with a lower pH value (Table 3). 

## Discussion

Urolithiasis formation is widely distributed, not only in Saudi Arabia but also globally. Multiple factors contribute to the development of urolithiasis, including hereditary factors, dietary habits, dehydration, climate variables, race, sex, age, occupation, and BMI. Understanding these modifiable factors in addition to stones composition can significantly contribute to decreasing the burden of renal calculi. For instance, the pathophysiology of urolithiasis requires an understanding of the composition of renal calculi.

Prior studies explored different aspects of kidney calculi in the Central Region of Saudi Arabia. For example, Khan et al. reported an increased male: female predominance, with a ratio of 5:1 [10]. Similarly, another study in the Eastern Region by Alkhunaizi et al. indicated that males were more frequently affected than females, with a male: female ratio of 3.9:1 [11]. In the current study, the ratio of male to female was 4:1, which is consistent with Khan et al. and Alkhunaizi et al. [10,11]. In contrast, studies conducted in Canada and the United State indicated a decline in the male to female ratio for the incidence of calculi [12-15].

The current study was conducted in the Eastern Region, which is known to be the hottest area in Saudi Arabia, with a temperature reaching up to 50°C. Dehydration, with subsequently concentrated urine, promotes an increase in urinary crystallization, supersaturation, and renal calculi formation. In this study, calcium oxalate was the most frequent type of calculi, followed by uric acid stone. This was congruent with the findings reported by Khan et al., Alkhunaizi et al., Amir et al., and Malik et al. [10,11,16,17].

The diversity in lifestyle in Saudi Arabia has led to a remarkable increase in the prevalence of obesity in several regions of the country. Weight abnormality has reached an epidemic level in some regions of the country where two-thirds of the Saudi citizens were overweight or obese. Obesity is usually associated with a high risk of diseases, including cardiovascular illness. Additionally, obesity is associated with an increased risk of renal calculi [18]. One study conducted in the United States concluded that obesity and weight gain were significantly associated with kidney stone formation [19]. Consistently, another study aimed to investigate the association between BMI, lipid profiles, and type of urinary calculi. Reported that the BMI was higher in stone formers, and it may be associated with different types of urinary calculi [20]. The current study revealed a positive association between BMI and renal calculi. This finding is consistent with the current literature [11,18].

For years, it has been recognized that a consistently low urine pH is linked to uric acid nephrolithiasis, despite the fact that its significance has just lately been recognized [21]. In the present study, we also found that a low urine pH was associated with uric acid stone formation, supporting the current literature [21,22].

We are aware that our research has some limitations, mainly related to the retrospective nature of the study with limited sample size. In addition, some critical information from the patient medical records was missing, such as diabetes mellitus, hypertension, and 24-hour urine collection tests. Our recommendation is that multi-centric studies with a large sample size in all the Saudi Arabian regions are required.

## Conclusions

The results of the current study indicated that the lifestyle of the residents based in this area, as well as the climatic nature of the region, contributed to the incidence of the stone disease; however, the small sample size and retrospective design limited this study. In addition, males are more affected by this disease, compared to females. Though the renal calculi were of different types, the calcium oxalate calculi were more prevalent in this region, compared to other types of renal calculi. These findings are consistent with the literature and they highlighted the necessity for further studies in this area, to provide insight into the pathophysiology and incidence of renal calculi for improving patient care.
